# Out of Pocket Costs for People with Dementia: A Comparison Across Insurance Types

**DOI:** 10.1093/geroni/igaf122.728

**Published:** 2025-12-31

**Authors:** Krista Harrison, Karen McKendrick, Lihua Li, Claire Ankuda, Lauren Hunt, Melissa Aldridge

**Affiliations:** University of California, San Francisco, San Francisco, California, United States; Icahn School of Medicine at Mount Sinai, New York, New York, United States; Icahn School of Medicine at Mount Sinai, New York, New York, United States; Icahn School of Medicine at Mount Sinai, New York, New York, United States; University of California, San Francisco, San Francisco, California, United States; Icahn School of Medicine at Mount Sinai, New York, New York, United States

## Abstract

People with dementia (PWD) comprise a significant proportion of enrollees in Traditional Medicare (TM), Medicare Advantage (MA), and Medicaid, with notable increases in MA. Although overall health differs by insurance, we know little about the types of healthcare driving out-of-pocket costs for people with dementia. We conducted a cohort study of PWD age 65+ in the Medicare Current Beneficiary Survey, 2002-2022. We categorized PWD based on 12 months of TM, MA, partial or full Medicaid dual enrollment. All analyses are survey-weighted and adjusted to 2022 dollars. Of 72,758 PWD, mean age was 75.7, 78.8% were White, 55.9% were female; 5.5% died. Mean annual out-of-pocket costs for PWD were $3,944; 31% due to outpatient costs, 20% prescriptions, 17% nursing homes, 17% assisted living and other facilities, 7% durable medical equipment, 3% inpatient costs, and 2% home health. Mean annual out-of-pocket costs were highest for PWD in TM ($4,590) compared to MA ($2,990) and Medicaid ($2,900). Despite differences in total out-of-pocket costs between TM and MA, proportional spending by healthcare type were similar, except for higher spending on prescriptions (MA 25% versus TM 20%). Among PWD with Medicaid, out-of-pocket costs for nursing homes and assisted living facilities were significantly higher (60%) compared to those TM (33%) and MA (30%). Although nursing home use declined among PWD using Medicaid, mean annual out-of-pocket costs increased from $10,950 in 2002 to $14,260 in 2022. PWD in TM spend the most in out-of-pocket costs; residential care for PWD continues to drive out-of-pocket spending across insurance types.

